# Associations Between Comorbidities, Medication Use and Dental Outcomes in Clinical Practice in Hail Dental Centre in Saudi Arabia: A Retrospective Study

**DOI:** 10.3290/j.ohpd.c_2615

**Published:** 2026-03-25

**Authors:** Abdulmajeed O. Alotaibi, Fahad Bakitian, Hatem D. Alshammari, Abdullah Alqhtani, Mohamed Omar Elboraey, Mostafa I Fayad, Ali Ghareeb Altamimi, Feras Mohammed Bokhamsin

**Affiliations:** a Abdulmajeed O. Alotaibi Assistant Professor, Department of Prosthodontics, Faculty of Dentistry, Taif University, Taif, Saudi Arabia. Study design, conducting the study, analysing the data, writing and reviewing the manuscript.; b Fahad Bakitian Associate Professor, Department of Restorative Dentistry, Faculty of Dental Medicine, Umm Al-Qura University, Makkah, Saudi Arabia. Study design, conducting the study, analysing the data, writing and reviewing the manuscript.; c Hatem D. Alshammari Professor, Department of Preventive Dentistry, College of Dentistry, University of Ha’il, Ha’il, Saudi Arabia. Study design, conducting the study, analysing the data, writing and reviewing the manuscript.; d Abdullah Alqhtani Professor, Department of Preventive Dental Sciences, College of Dentistry, Taibah University, Medina, Saudi Arabia. Study design, conducting the study, analysing the data, writing and reviewing the manuscript.; e Mohamed Omar Elboraey Researcher, Department of Preventive Dental Sciences, College of Dentistry, Taibah University, Medina, Saudi Arabia. Study design, conducting the study, analysing the data, writing and reviewing the manuscript.; f Mostafa I Fayad Associate Professor, Department of Substitutive Dental Science, College of Dentistry, Taibah University, Medina, Saudi Arabia. Study design, conducting the study, analysing the data, writing and reviewing the manuscript.; g Ali Ghareeb Altamimi Consultant in Prosthodontics, Ministry of Health, Riyadh, Saudi Arabia. Study design, conducting the study, analysing the data, writing and reviewing the manuscript.; h Feras Mohammed Bokhamsin Dentist, Ministry of Health, Riyadh, Saudi Arabia. Study design, conducting the study, analysing the data, writing and reviewing the manuscript.

**Keywords:** chronic diseases, dental care, dental caries, medically compromised patients, polypharmacy, risk assessment

## Abstract

**Background:**

Patients with medical conditions present unique challenges in dental practice, requiring modifications to treatment protocols and heightened awareness of potential complications. Understanding the prevalence and patterns of medical conditions and medication use among dental patients is crucial for providing safe and effective care.

**Purpose:**

To examine the prevalence and patterns of medical conditions and medication use among dental patients, and to explore possible links between medical conditions and dental health outcomes.

**Methods and Materials:**

This retrospective study was conducted on 383 dental patients in Hail, Saudi Arabia. Demographic information, medical history, medication profiles, and dental health parameters were analysed. Descriptive statistics were used to characterise the sample and associations between medical conditions, medications, and dental health outcomes.

**Results:**

The mean age of patients was 51.5 years (SD = 16.5), with a female predominance (65%). The prevalence of diabetes was 53.5%, hypertension 49.6%, and hypothyroidism 23.0%. Polypharmacy (≥ 5 medications) occurred in 8.4% of patients. Commonly used medications included antihypertensives, antidiabetics, and analgesics. The mean number of active dental caries was 1.37 per patient. Significant associations were observed between diabetes and the number of active caries, and hypertension was associated with increased dental pain.

**Conclusions:**

The findings highlight the high prevalence of medical conditions and medication use among dental patients, emphasising the importance of comprehensive medical history assessment in dental practice. Understanding these patterns can inform treatment planning, risk assessment, and preventive strategies for medically compromised dental patients.

As the prevalence of systemic diseases continues to rise, dentists are increasingly encountering patients with complex medical histories and medication regimens. Consequently, the link between oral health and systemic diseases has become a central focus in both dental research and clinical practice. Recent epidemiological data from the World Health Organization indicate that untreated dental caries is among the most common health conditions worldwide, affecting over 2.3 billion people. Moreover, severe periodontal disease impacts approximately 10% of the global population. At the same time, chronic conditions such as diabetes (537 million) and hypertension (1.28 billion) are also increasing, reshaping how dental treatments are planned and delivered.^17^ Individuals with chronic or systemic conditions that influence their response to dental treatment require customised clinical approaches to ensure safety and effectiveness. These patients often present with cardiovascular disease, diabetes, respiratory disorders, immunodeficiencies, and other comorbidities, many of which are managed with medications that can adversely affect oral health, leading to drug interactions, xerostomia, or delayed healing.^12^

Recent investigations have further elucidated the bidirectional connections between oral and systemic health. Natarajan et al (2025)^13^ reported moderate associations between periodontitis and diabetes (Cramer’s V = 0.14) and between dental caries and hypertension (Cramer’s V = 0.12). In a multicountry analysis, the prevalence of systemic conditions among dental patients ranged from 28.2% to 88.7%, influenced by age, gender, socioeconomic status, and care setting. Notably, 39.2% of dental patients aged ≥ 65 years were taking five or more medications, indicating a high prevalence of polypharmacy.^8^ These findings emphasise the substantial burden of comorbidities and medication use within dental populations. Singh and Papas (2024)16 demonstrated the oral consequences of polypharmacy in geriatric patients, noting that more than 400 commonly prescribed medications can induce xerostomia, significantly increasing the risk of dental caries, periodontal disease, and oral infections. The authors identified anticholinergics, antidepressants, antihypertensives, antihistamines, and diuretics as the pharmacological classes most frequently associated with hyposalivation, underscoring the need for thorough medication review and salivary assessment in dental care. Complementing these findings, a recent umbrella analysis synthesised data from 32 systematic reviews and provided strong evidence for bidirectional relationships between periodontitis and diabetes mellitus. Specifically, periodontitis was associated with impaired glycemic control, whereas diabetes increased both the risk and severity of periodontal disease.^15^

Despite the growing awareness of the critical importance of medical considerations in dental practice, several challenges continue to hinder the delivery of safe and effective care to medically compromised patients. A recent qualitative study identified multiple barriers that limit optimal management in such cases, including insufficient knowledge of systemic disease interactions, inadequate training in pharmacological management, limited access to comprehensive medical records, and suboptimal communication between dental and medical professionals.^14^ These barriers are often compounded by significant time constraints in clinical settings, which reduce opportunities for thorough patient assessment and interdisciplinary collaboration. Collectively, these findings underscore the urgent need for improved integration of medical and dental education, as well as structured continuing professional development programmes to enhance clinicians’ competency in managing complex patients.^14^ While substantial research has investigated the prevalence of systemic diseases and medication use among dental patients, the direct associations between these factors and specific oral health outcomes remain insufficiently characterised. This knowledge gap is particularly evident for less common systemic conditions and newer classes of pharmacological agents, where empirical data are limited.^5,11^ Furthermore, regional variations in population health profiles, healthcare infrastructure, and prescribing patterns complicate the generalisation of international findings to local contexts.

In Saudi Arabia, emerging evidence suggests a substantial burden of systemic diseases among patients seeking dental care. Recent studies conducted in teaching hospitals and public dental clinics have reported high prevalence rates of diabetes, hypertension, and other chronic conditions among dental attendees, often accompanied by complex medication regimens.^1,2,3^ These studies have also highlighted challenges related to oral health practices, access to care, and treatment barriers in medically compromised patients. However, most Saudi-based investigations have focused primarily on describing the prevalence of medical conditions or assessing patient awareness, rather than examining the direct relationships between systemic diseases, medication burden, and specific oral health outcomes. Consequently, despite the growing recognition of the importance of medical considerations in dental practice, locally relevant data linking comorbidities and pharmacological therapy to adverse dental outcomes remain limited. As a result, there is a clear need for large-scale, population-based investigations that capture the epidemiological patterns of systemic disease, medication use, and their impact on oral health within the Saudi population. The primary research question was whether systemic comorbidities and medication burden are associated with adverse oral health outcomes among dental patients in Saudi Arabia. Accordingly, the present study aimed to: (1) determine the prevalence and trends of systemic diseases and medication use among dental patients; (2) analyse the demographic characteristics and trends of patients presenting with systemic illnesses and pharmacological therapies; and (3) identify key risk factors associated with adverse oral health outcomes in medically compromised populations.

## METHODS AND MATERIALS

### Study Design, Data Collection, and Ethical Approval

This retrospective cross-sectional study analysed data from 383 dental patients referred by general dental practitioners to Hail Dental Centre. The data were obtained from patient records of individuals who attended dental screening clinics between November and December 2024. The sample size was determined by the number of eligible patient records available during the study period. As this was a retrospective record-based study, *a priori* power analysis was not performed. All consecutive eligible records were included to maximise representativeness.

#### Inclusion criteria

Adult patients (≥18 years) attending Hail Dental Centre during the study period with documented medical history, medication use, and dental outcomes.

#### Exclusion criteria

Records with missing key variables, duplicate visits (only the first visit included), or charts lacking a clinical dental assessment. Patient records included comprehensive information encompassing demographic details, medical histories, medication profiles, and dental health parameters. Medical conditions were categorised by body system (eg, cardiovascular, endocrine, respiratory) and specific diagnoses, following the World Health Organization’s current medical taxonomy.^15,17^ Medications were classified into therapeutic categories based on contemporary pharmacological literature.^16^ Dental health parameters were derived from clinical examination findings recorded in the patient files.

Primary variables of interest included:

Demographic characteristics: Included age, gender, and referral source, categorised according to epidemiological research standards.^8^
Medical conditions: Included diabetes, hypertension, asthma, rheumatoid arthritis, hypothyroidism, cardiac disorders, stroke, and other relevant diseases.^14^
Medications: Classified into major pharmacological groups such as antihypertensives (eg, beta-blockers), antidiabetics (eg, insulin, metformin), analgesics (eg, NSAIDs), and thyroid medications (eg, levothyroxine), according to standard pharmacological references.
Polypharmacy: Defined as the total number of medications per patient, categorised as none (0), low (1–2), moderate (3–4), and high (≥ 5), following current dental and medical literature.^16^
Dental health parameters: Dental outcomes were limited to dental pain and caries measures, as these variables were consistently and uniformly documented across patient records. Other oral health parameters – including periodontal status, oral mucosal conditions, dental restorations, and edentulism – were not recorded in a standardised manner and were therefore excluded to minimise information bias. Dental pain and caries were selected due to their clinical relevance and suitability for retrospective analysis.^13^

Referrals were primarily received from primary healthcare centres (PHCs) and hospitals, consistent with patterns reported in previous epidemiological studies.^8^ Data extraction was performed using a standardised data collection form, following established methodologies for retrospective dental research.^14^ Furthermore, this study adhered to international ethical principles for research involving human subjects. Patient confidentiality was ensured by anonymising all data prior to analysis and removing personal identifiers. Ethical approval was granted by the Taibah University College of Dentistry Research Ethics Committee (Approval No. TUCDREC/070933/AAlqhtani).

### Statistical Analysis

A combination of descriptive, inferential, and machine learning techniques was employed to analyse the data.

#### Descriptive statistics

Basic demographic and clinical characteristics were summarised using means, standard deviations, ranges, and proportions. These included age distribution, gender, prevalence of systemic diseases, medication usage patterns, and dental health parameters.

#### Correlation analysis

Pearson correlation coefficients were computed to examine linear relationships between variables, including:

Age and number of comorbidities
Number of medications and number of active caries
Pairwise associations between systemic conditions (eg, diabetes and hypertension)

#### Comparative analysis

Group differences were evaluated using:

Independent samples t-tests for continuous variables (eg, mean number of caries between diabetic and non-diabetic patients)
Chi-square tests for categorical variables (eg, prevalence of dental pain across hypertensive vs non-hypertensive groups)

#### Multivariate regression analysis

A multiple linear regression model was applied to identify independent predictors of caries count. Covariates included age, diabetes, hypertension, and hypothyroidism. Beta coefficients (β) and P values were reported to determine the strength and significance of associations.

#### Cluster and network analysis

Cluster analysis was used to identify distinct patient profiles based on age, number of medications, and caries count. Network analysis was subsequently performed to explore interrelationships among prevalent systemic conditions within the study cohort.

#### Decision tree modelling

A decision tree model was developed to predict the presence of active caries using five predictors: age, diabetes, hypertension, hypothyroidism, and total number of medications. Model performance was evaluated using accuracy, precision, recall, and F1-score metrics. Feature importance was analysed to identify the most influential predictors, and decision rules were interpreted from the resulting tree structure.

## RESULTS

### Demographic Characteristics

A total of 383 dental patients were included in the analysis, with a mean age of 51.5 ± 16.5 years (range: 16–95 years). The majority were female (65.0%), while males accounted for 35.0% of the sample (Table 1). The primary source of referrals was primary health centres (PHCs) (58.2%), followed by hospitals or speciality clinics (41.8%). Patients were categorised into four age groups: < 35 years (27.2%), 36–50 years (29.2%), 51–65 years (27.9%), and > 65 years (15.7%) (Table 1).

**Table 1:** Demographic characteristics and referral sources of the study population (n = 383)

Variable	Category	n	%	Mean ± SD/Range	P value
Age (years)	–	–	–	51.5 ± 16.5 (16–95)	–
Gender	Male	134	35.0	–	–
	Female	249	65.0	–	–
Referral source	Primary health centre	223	58.2	–	–
	Hospital/speciality clinic	160	41.8	–	–
Age group (years)	< 35	104	27.2	–	–
	36–50	112	29.2	–	–
	51–65	107	27.9	–	–
	> 65	60	15.7	–	–


### Prevalence of Medical Conditions

**Fig 1:**
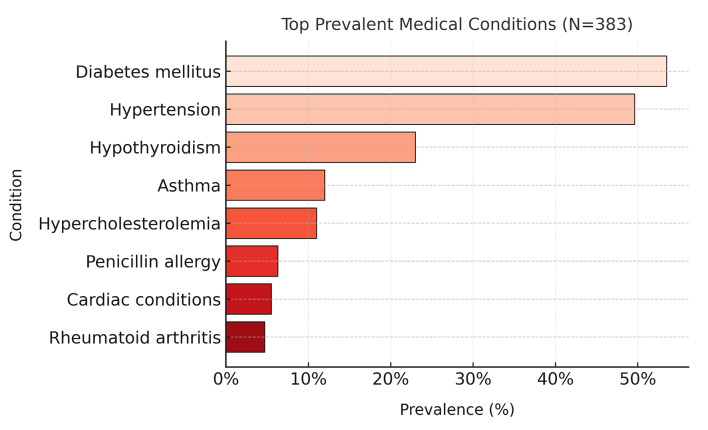
Prevalence of major medical conditions among dental patients.

**Fig 2:**
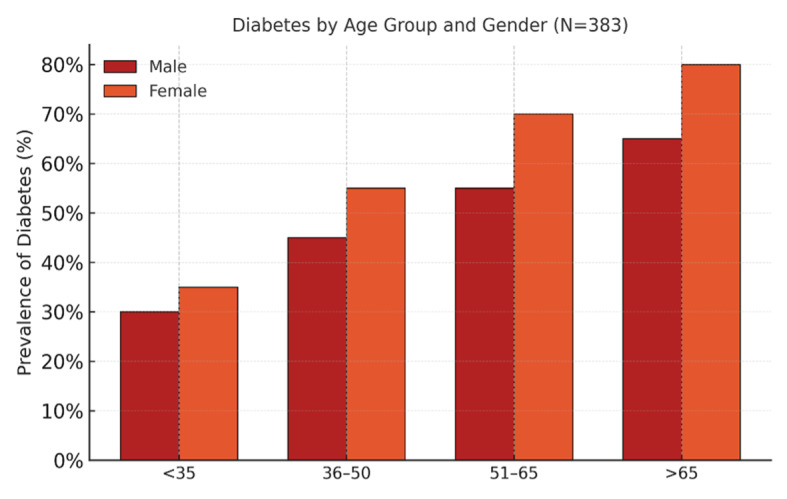

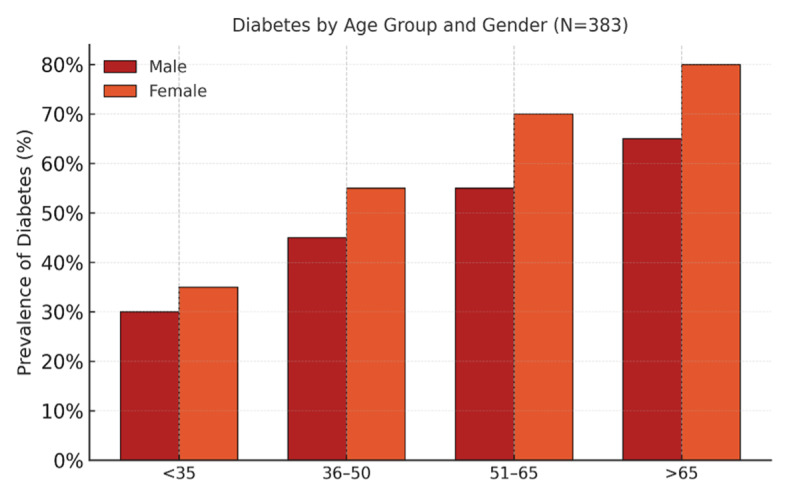

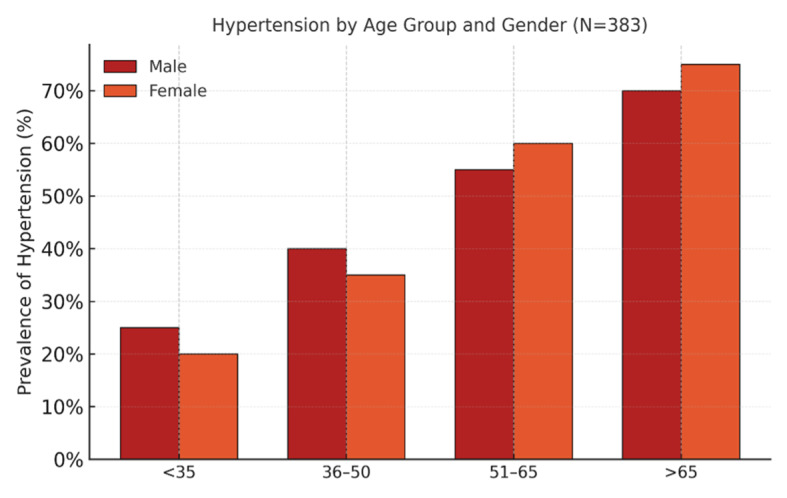
Distribution of medical conditions by age and gender (diabetes, hypertension, hypothyroidism).

Overall, 76.2% of patients presented with at least one systemic medical condition. The most prevalent conditions were diabetes mellitus (53.5%), hypertension (49.6%), and hypothyroidism (23.0%) (Table 2, Fig 1). Age and gender significantly influenced these three medical condition distributions (Fig 2). Diabetes and hypertension were more frequent among older adults (P < 0.001 for both), while hypothyroidism showed a strong female predominance (89.0% female, P = 0.032). Other conditions included asthma (12.0%), hypercholesterolemia (11.0%), cardiac disease (5.5%), rheumatoid arthritis (4.7%), stroke or neurological disease (3.1%), and chronic kidney disease (2.1%) (Table 2).

**Table 2:** Prevalence of medical conditions among dental patients

Medical condition	n	%	Mean age ± SD	Female %	P (age)
Diabetes mellitus	205	53.5	56.2 ± 11.4	62.4	< 0.001
Hypertension	190	49.6	58.7 ± 9.8	59.3	< 0.001
Hypothyroidism	88	23.0	52.8 ± 12.1	89.0	0.032
Asthma	46	12.0	49.1 ± 14.6	68.3	0.211
Hypercholesterolemia	42	11.0	58.9 ± 10.5	52.4	0.028
Cardiac disease	21	5.5	61.3 ± 8.2	47.6	0.015
Rheumatoid arthritis	18	4.7	54.5 ± 10.9	83.3	0.091
Stroke / Neurological disease	12	3.1	63.7 ± 9.5	41.7	0.046
Chronic kidney disease	8	2.1	60.9 ± 7.3	62.5	0.128


Multiple comorbidities were frequent: 42.3% of patients had two or more systemic diseases. The number of comorbidities correlated positively with age (r = 0.42, P < 0.001). Patients aged > 65 years exhibited an average of 2.87 comorbidities, compared with 0.82 among those < 35 years.

### Medication Use Patterns

**Fig 3:**
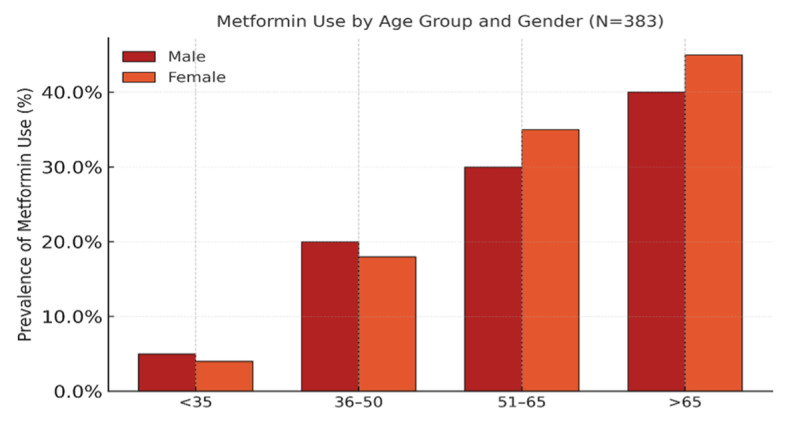

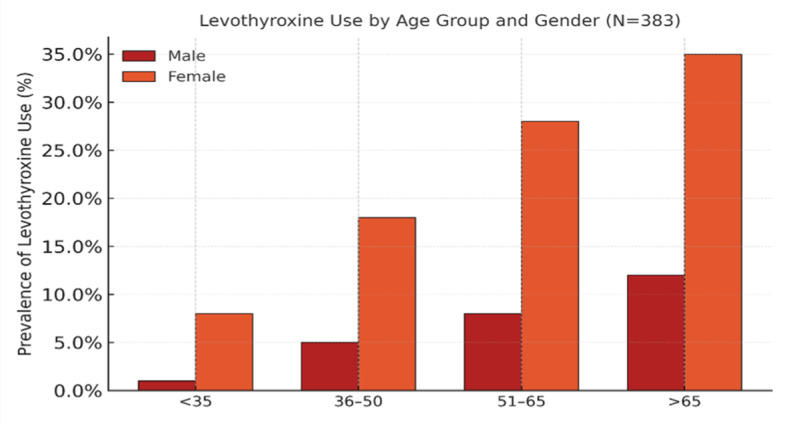
Distribution of levothyroxine use by age and gender.

The mean number of medications per patient was 2.24 ± 1.8 (range: 0–8). The most frequently used drug classes were beta-blockers (49.1%), metformin (33.7%), levothyroxine (24.8%), and insulin (21.9%) (Table 3; Fig 3). Other commonly prescribed medications included aspirin (13.6%), non-steroidal anti-inflammatory drugs (NSAIDs) (11.0%), and albuterol (10.2%).

**Table 3:** Medication use patterns and prevalence of polypharmacy

Medication use	n	%	Mean no. drugs ± SD	Associated condition	P value
Beta-blockers	188	49.1	2.8 ± 1.5	Hypertension	0.007
Metformin	129	33.7	2.6 ± 1.4	Diabetes mellitus	< 0.001
Levothyroxine	95	24.8	2.3 ± 1.3	Hypothyroidism	0.092
Insulin	84	21.9	3.1 ± 1.7	Diabetes mellitus	< 0.001
Aspirin	52	13.6	2.7 ± 1.6	Cardiovascular	0.051
NSAIDs	42	11.0	2.4 ± 1.2	Musculoskeletal pain	0.314
Albuterol	39	10.2	2.1 ± 1.1	Asthma	0.201
Polypharmacy ≥ 5 meds	32	8.4	–	–	–
Low (1–2 meds)	148	38.5	–	–	–
Moderate (3–4 meds)	115	30.0	–	–	–
None (0 meds)	88	23.1	–	–	–


Polypharmacy, defined as the use of five or more medications, was identified in 8.4% of patients. The distribution of medication use was as follows: none (23.1%), low (1–2 drugs, 38.5%), moderate (3–4 drugs, 30.0%), and high (≥ 5 drugs, 8.4%).

Medication use increased significantly with age (P < 0.001), with older adults (> 65 years) averaging 3.12 medications, compared to 0.75 in those younger than 35 years. Among therapeutic classes, beta-blocker use correlated significantly with hypertension (P = 0.007), and antidiabetic medications (metformin and insulin) were strongly linked to diabetes (both P < 0.001).

### Dental Health Parameters

Dental pain was reported in 58.7% of patients, and active caries were detected in 68.9%. The mean number of active caries was 1.37 ± 2.1 (range: 0–28), with a right-skewed distribution; most patients presented with one or two carious lesions, while a small subset exhibited extensive caries involvement (Tables 4 and 5).

**Table 4:** Association between medical conditions and dental health parameters

Medical condition	Dental pain (%)	Active caries (%)	Mean no. of caries ± SD	P (pain)	P (caries presence)	P (mean caries)
Diabetes mellitus	61.5	78.5	1.92 ± 2.3	0.183	0.003 *	< 0.001 *
Hypertension	67.4	65.3	1.41 ± 2.0	0.024 *	0.271	0.213
Hypothyroidism	59.8	71.6	1.58 ± 2.2	0.612	0.182	0.092
Asthma	56.3	64.1	1.28 ± 1.9	0.812	0.312	0.375
Rheumatoid arthritis	62.5	68.8	1.47 ± 2.1	0.928	0.531	0.522
Cardiac disease	64.7	63.2	1.25 ± 1.8	0.884	0.451	0.612


**Table 5:** Association between medication use and dental health parameters

Medication	Dental pain (%)	Active caries (%)	Mean caries ± SD	r	P (pain)	P (caries)
Beta-blockers	69.1	67.8	1.43 ± 2.0	0.31	0.007 *	0.151
Metformin	60.3	76.4	1.87 ± 2.1	0.29	0.111	< 0.001 *
Insulin	62.5	82.2	2.37 ± 2.6	0.35	0.084	< 0.001 *
Levothyroxine	55.8	71.6	1.58 ± 2.2	0.18	0.231	0.092
Aspirin	58.9	69.2	1.41 ± 2.1	0.27	0.154	0.313
NSAIDs	54.7	66.7	1.26 ± 1.9	0.14	0.335	0.274
Albuterol	59.0	68.0	1.35 ± 2.0	0.19	0.401	0.362


### Associations Between Medical Conditions, Medication Use Patterns, and Dental Health Parameters

Statistical analyses revealed several significant associations between medical conditions, medication use, and dental health parameters (Tables 4 and 5). Diabetes mellitus was strongly associated with both the presence (78.5% vs 58.0%, P = 0.003) and number of active caries (1.92 ± 2.3 vs 0.73 ± 1.6, P < 0.001). Hypertension was linked to increased dental pain (67.4% vs 50.0%, P = 0.024). Hypothyroidism showed a non-significant trend towards a higher caries count (1.58 vs 1.31, P = 0.092).

The number of medications correlated positively with the number of active caries (r = 0.31, P < 0.001). Patients on beta-blockers reported greater dental pain (69.1% vs 48.5%, P = 0.007), while those using insulin had a higher mean caries count (2.37 ± 2.6 vs 1.08 ± 1.9, P < 0.001) (Table 5).

### Multivariate Regression Analysis

In the multivariate linear regression model (Table 6a), age showed a negative association with caries count (β = −0.024, P = 0.004), indicating fewer caries among older individuals when controlling for systemic diseases. Diabetes mellitus emerged as an independent predictor of caries count (β = 1.193, P < 0.001), while hypertension (β = 0.025, P = 0.935) and hypothyroidism (β = 0.291, P = 0.428) were non-significant.

**Table 6a and b:** Multivariate regression models for predictors of dental caries

(a) Linear regression – dependent variable: number of active caries				
Predictor	β	SE	t	P
(b) Logistic regression — dependent variable: presence of active caries (yes/no)				
Predictor	OR (95% CI)		P	
Age (years)	−0.024	0.008	−2.91	0.004 *
Diabetes mellitus	1.193	0.273	4.37	< 0.001 *
Hypertension	0.025	0.290	0.08	0.935
Hypothyroidism	0.291	0.364	0.80	0.428
Age (years)	1.080 (1.050–1.110)		< 0.001 *	
Diabetes mellitus	0.193 (0.058–0.644)		0.007 *	
Hypertension	0.199 (0.058–0.685)		0.010 *	
Hypothyroidism	1.021 (0.624–1.735)		0.921	
Model fit: Adjusted R^2^ = 0.192; F = 8.67; P < 0.001.				
Model fit: Pseudo R^2^ = 0.302; P < 0.001				

**Fig 4:**
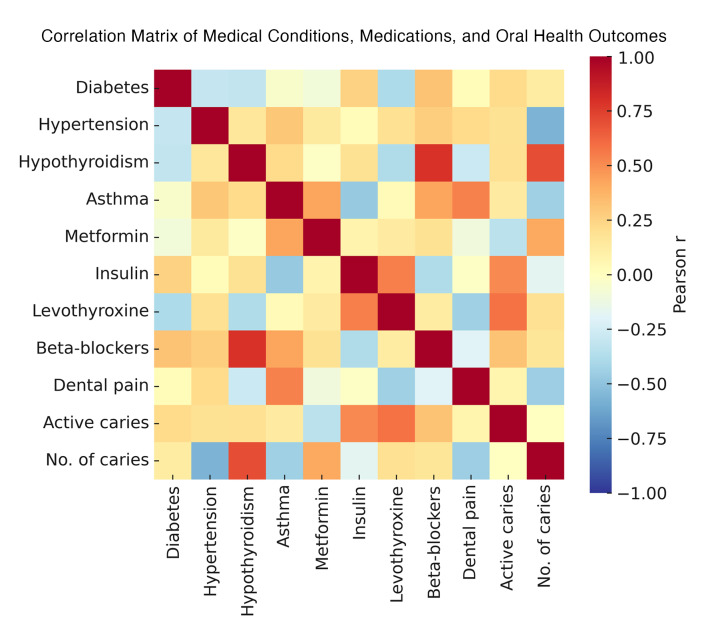
Correlation matrix illustrates relationships among medical conditions, medications, and dental outcomes.

The logistic regression model (Table 6b) confirmed these findings: diabetes (OR = 0.193, 95% CI = 0.058–0.644, P = 0.007) and hypertension (OR = 0.199, 95% CI = 0.058–0.685, P = 0.010) significantly influenced the likelihood of having active caries. These interrelationships between systemic diseases, medication use, and oral health outcomes are illustrated in Figure 4.

### Cluster and Network Analyses

Cluster analysis identified three distinct patient groups (Table 7):

**Table 7:** Multivariate analyses exploring demographic, clinical, and systemic health predictors of disease patterns

Analysis type	Main variables	Key findings/metrics	Interpretation
K-means clustering	Age, no. of medications, caries count	3 clusters identified: C0 older ( n ≈ 210 ); C1 younger ( n ≈ 165 ); C2 outlier ( n ≈ 8 )	Distinct age-related profiles of disease burden and caries experience
Network analysis	Diabetes, hypertension, hypothyroidism, asthma	Strongest links: DM–HTN r = 0.275; HTN–Hypo r = 0.155; DM–Hypo r = 0.104	Suggests shared cardiometabolic risk pathways
Decision tree model	Age, diabetes, hypertension, no. of medications	Accuracy = 74.4%; precision = 75%; recall = 85.7%; f1 = 80%; primary split age = 42.5 y	Age dominant predictor (65.1%), followed by diabetes (19.8%) and medication count (15.1%)

Cluster 0: Older patients (mean age = 53.1 years) with moderate medication use (1.8 drugs) and moderate caries (1.4 lesions); high prevalence of diabetes (48.6%) and hypertension (40.5%).
Cluster 1: Younger patients (mean age = 28.5 years) with minimal medication use (1.0) and low caries levels (0.9); minimal systemic disease prevalence.
Cluster 2: Small outlier group characterised by extreme caries burden.

**Fig 5:**
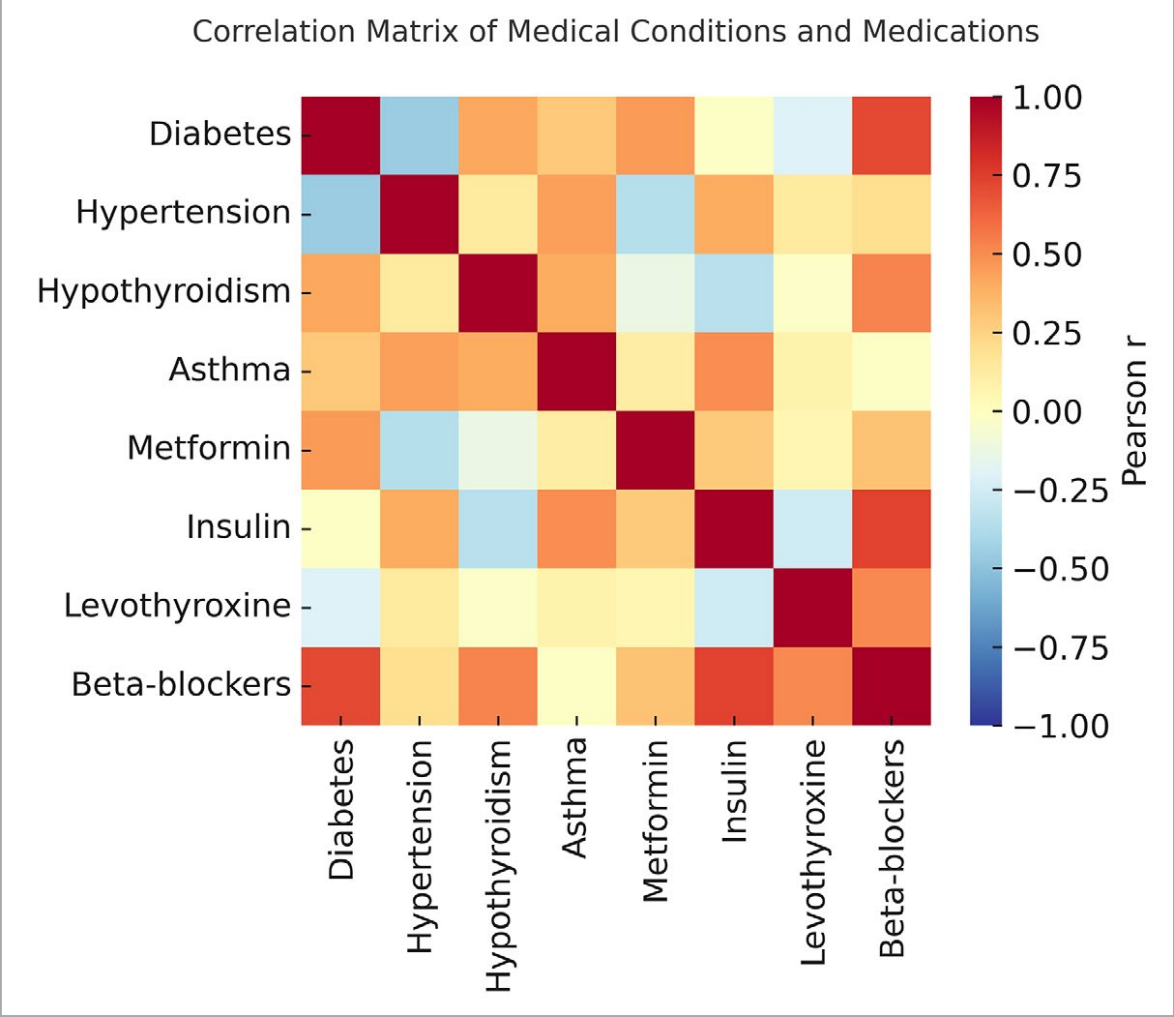
Correlation matrix of medical conditions and medication use.

Network analysis revealed the strongest comorbidity link between diabetes and hypertension (r = 0.275), followed by hypertension and hypothyroidism (r = 0.155) and diabetes and hypothyroidism (r = 0.104) (Table 7, Fig 5). Asthma exhibited minimal correlations, reflecting distinct pathophysiological mechanisms.

### Decision Tree Model Analysis

The decision tree model demonstrated strong predictive performance (accuracy = 74.4%, precision = 75.0%, recall = 85.7%, F1 = 80.0%). Feature importance analysis identified age (65.1%), diabetes (19.8%), and medication count (15.1%) as the most influential predictors (Table 7; Fig 5).

The model’s primary split occurred at age = 42.5 years. Among younger patients (< 42.5 years), diabetes status determined caries risk, while among older patients (≥ 42.5 years), medication burden was the dominant stratifier.

### Gender Considerations

Gender-based differences were observed for specific systemic diseases. Hypothyroidism was significantly more prevalent among females (32.7%) than males (3.6%) (P < 0.001). Conversely, cardiovascular diseases were more frequent in males (57.1%) compared with females (46.2%) (P = 0.048). No significant gender differences were identified in medication use patterns or dental health parameters.

## DISCUSSION

This study examined the prevalence and patterns of medical conditions and medication use among dental patients, as well as the links between health status and dental outcomes. The results showed a high rate of medical conditions, with diabetes mellitus, hypertension, and hypothyroidism being the most common. Significant polypharmacy was observed, with beta-blockers, metformin, and insulin among the most used medications. The findings also revealed several essential associations between medical conditions, medication use, and dental health parameters.

The high rate of medical conditions observed in this study (76.2% of patients with at least one condition) aligns with previous research findings. Fernández-Feijoo et al (2012) reported that 28.2% to 88.7% of dental patients had at least one systemic disease, with significant variation across different healthcare settings and populations.^8^ Our finding of 76.2% falls within this range. However, it is on the higher end, possibly reflecting the characteristics of our patient population, which included many referrals from PHCs and hospitals.

The high prevalence rates of diabetes mellitus, at 53.5%, and hypertension, at 49.6%, observed in our sample are notable and align with the broader, well-documented global trends indicating increasing rates of chronic diseases worldwide. However, these rates are considerably higher compared to those reported in some previous research studies. For instance, a study conducted by Samara et al (2024)^14^ reported a prevalence of diabetes among dental patients that ranged from as low as 8.5% to 15.2%, which is markedly lower than the 53.5% observed in our sample. Such discrepancies could be attributed to various factors, including regional differences in disease prevalence – some regions naturally exhibit higher rates due to genetic, environmental, or lifestyle factors – as well as differences in the characteristics of studied populations, such as age, socioeconomic status, or access to healthcare services. Additionally, there is the possibility of selection bias in our sample, which might have disproportionately included individuals with higher health risks or existing conditions. Furthermore, according to the World Health Organization (WHO) 2023 global health statistics, the estimated prevalence of diabetes in the adult population worldwide is approximately 10.5%. This comparison suggests that our sample may constitute a subset of a population experiencing a particularly elevated burden of these chronic conditions, possibly due to geographic, demographic, or clinical factors specific to the study site.^17^

The high prevalence of hypothyroidism (23.0%) in our study is especially noteworthy and exceeds rates reported in general population studies. Natarajan et al (2025) found a hypothyroidism prevalence of 4.6% to 9.5% in their cross-sectional analysis of dental patients.^13^ The elevated rate in our sample may reflect regional iodine deficiency, higher rates of autoimmune disorders, or demographic factors such as the predominance of females, who are more susceptible to thyroid disorders.^6^ The pattern of multiple comorbidities observed in our study (42.3% with two or more conditions) demonstrates the growing challenge of multimorbidity in healthcare. Seitz et al (2019) highlighted the increasing prevalence of multiple chronic conditions, particularly among older adults, and emphasised the complex interactions between these conditions and their implications for dental care.^15^ Our findings reinforce the importance of comprehensive medical assessment and integrated care approaches for dental patients. The medication use patterns observed in our study have important implications for dental practice. The mean number of medications per patient (2.24) and the significant proportion of patients with polypharmacy (8.4% taking ≥ 5 medications) highlight the potential for drug interactions and medication-related oral side effects. Kim et al (2024) reported polypharmacy rates of 39.2% among dental patients aged 65 and older, which is considerably higher than our overall rate but potentially comparable to our rates among older age groups.^9^

The notable links between certain medical conditions and dental health parameters offer insights into the complex relationship between systemic and oral health. The higher prevalence and severity of dental caries observed among diabetic patients in the present study (78.5% vs 58.0%, P = 0.003) is consistent with established evidence linking diabetes to adverse oral health outcomes. Comprehensive reviews by Leite et al^10^ have highlighted that individuals with diabetes are particularly vulnerable to oral complications, including dental caries, especially in the presence of salivary dysfunction and coexisting systemic conditions. Similarly, Seitz et al described several biological mechanisms underlying the diabetes–caries relationship, including impaired salivary flow and composition, alterations in oral microbiota, compromised immune response, and the effects of advanced glycation end products on collagen metabolism and tissue repair.^15^ Together, these mechanisms provide a plausible biological explanation for the associations observed in the current cohort and support the notion that metabolic dysregulation and medication-related effects may synergistically contribute to increased caries susceptibility in patients with diabetes. Variations in the magnitude of reported associations across studies may reflect differences in study design, population characteristics, disease control, and healthcare delivery settings. On the other hand, the link between hypertension and dental pain (67.4% vs 50.0%, P = 0.024) is a compelling finding that requires further exploration. Although the exact mechanism is not fully understood, Aguilera et al (2021) suggested several possible explanations, including shared inflammatory pathways, vascular changes affecting pulpal blood flow, and the potential effects of antihypertensive medications on pain perception.^12^ Our results contribute to the growing body of evidence on the connections between cardiovascular health and oral health, emphasising the importance of integrated patient care strategies.

The advanced statistical analyses performed in this study offer deeper insights into the complex relationships between medical conditions, medications, and dental health outcomes. The multivariate regression analysis confirmed the significant link between diabetes and dental caries (β = 1.193, P < 0.001), even after adjusting for age and other medical conditions. This strong finding reinforces the evidence that diabetes is an independent risk factor for dental caries, supporting the development of targeted preventive strategies for diabetic patients.

The negative association between age and the number of active caries in the multivariate model (β = -0.024, P = 0.004) is notable. It indicates that older age may serve as a protective factor against caries when controlling for medical conditions. This result aligns with the age-related trends seen in our descriptive analysis, where caries prevalence peaked in middle age and decreased in older groups. As suggested by Natarajan et al (2025), this pattern may be due to higher rates of edentulism, restored teeth, or different dietary habits among elderly patients.^13^

The cluster analysis conducted in this study provided a comprehensive classification of patient profiles by examining variables such as age, medication use, and caries experience. The analysis clearly delineated two primary groups: older patients with multiple comorbidities and younger, healthier individuals. This distinction emphasises the importance of developing age-specific dental care strategies tailored to the unique needs of each group. These findings lend empirical support to the patient stratification concept proposed by Samara et al (2024), which advocates for personalised medicine approaches in dental treatment.^14^ Moreover, the network analysis of comorbidities uncovered significant associations between conditions like diabetes, hypertension, and hypothyroidism. These links suggest potential shared underlying biological mechanisms or common risk factors, aligning with the concept of ‘cardiometabolic syndrome’ as detailed by WHO (2000).^7^ The network approach employed in this research offers both a visual representation and a quantitative framework for understanding the interconnected nature of these conditions. This methodology could be instrumental in identifying patients who are at increased risk for developing multiple comorbidities, thereby enabling earlier intervention and more targeted preventive measures. The decision tree analysis showed that age is the main predictor of active caries, making up 65.1% of the model’s predictive ability. This emphasises the need for age-specific strategies in caries risk assessment and prevention. The secondary factors, diabetes (19.8%) and the number of medications (15.1%), demonstrate how these variables influence caries risk and support their inclusion in risk assessment protocols.

Notably, our findings add to the growing evidence of the bidirectional links between oral and systemic health. The connections between diabetes and dental caries, hypertension and dental pain, and polypharmacy and oral health outcomes reveal complex interactions that may involve shared risk factors, biological processes, and healthcare utilisation patterns. Therefore, the high prevalence of medical conditions and medication use among dental patients has important implications for clinical practice. Taking a thorough medical history, evaluating risk, and adjusting treatments are essential for providing safe and effective dental care to patients with pre-existing medical conditions.

The connections identified in our study between specific medical conditions, such as diabetes and cardiovascular diseases, medications, including insulin and beta-blockers, and various dental health measures, offer valuable insights for developing targeted prevention strategies. For instance, our findings indicate that diabetic patients and those using insulin are at a notably higher risk of developing dental caries. This underscores the importance of implementing more comprehensive preventive measures for these populations, such as scheduling more frequent recall visits to monitor oral health, applying professional fluoride treatments to strengthen enamel, and providing tailored dietary counselling to reduce sugar intake and promote oral hygiene. The association between medication burden and adverse dental outcomes observed in the present study – particularly higher caries prevalence and increased dental pain – supports the role of polypharmacy as an important modifier of oral health in medically compromised patients. This finding is consistent with recent evidence highlighting polypharmacy as a major contributor to oral disease, particularly among medically compromised and older populations. A recent comprehensive review by Alshahrani et al^4^ emphasised that polypharmacy is strongly associated with xerostomia, dental caries, periodontal disease, and oral mucosal lesions through cumulative pharmacological effects on salivary secretion, immune modulation, and bone metabolism. The authors further noted that commonly prescribed drug classes – including antihypertensives, antidiabetic agents, antidepressants, and anticholinergics – exert additive or synergistic effects that increase vulnerability to caries and complicate dental management. These observations align with the current findings and support the interpretation that medication burden acts as an independent and modifiable risk factor for poor dental outcomes in secondary care settings. Differences in reported effect sizes across studies may reflect variations in patient age, comorbidity profiles, medication regimens, and access to preventive dental care. Additionally, the present study identified a significant association between beta-blocker use and increased reports of dental pain. This highlights the necessity for dental practitioners to incorporate thorough pain assessment protocols and develop customised pain management plans for patients on beta-blockers, ensuring better pain control and overall oral health outcomes. Recognising these interconnected factors allows healthcare providers to collaborate more effectively, offering integrated care that addresses both medical and dental health needs for at-risk patient populations.

Although this study has several strengths, such as the comprehensive assessment of medical conditions, medication use, and dental health parameters in a diverse patient population, some limitations must be acknowledged. For instance, the retrospective cross-sectional design prevents establishing causal relationships and analysing changes over time. Additionally, while the sample size is sufficient for many studies, it might limit the statistical power to identify associations with less common conditions or medications. Relying on clinical records could introduce information bias, especially for variables that require standardised assessment or are poorly documented. The single-centre nature of the study may also limit the ability to generalise the findings to other settings or populations. Finally, potential confounders, including socioeconomic status, dietary habits, tobacco use, oral hygiene practices, and access to dental care, were not available in the records and therefore could not be adjusted for in the analyses. Future studies should employ prospective, multicenter designs across different Saudi regions, incorporate standardised periodontal and oral hygiene indices, and include behavioural and socioeconomic variables. Such approaches would allow more comprehensive risk modelling and support evidence-based oral health planning.

## CONCLUSION

In Hail Dental Centre, systemic comorbidities, particularly diabetes and medication burden, were associated with unfavourable dental outcomes. These findings highlight the need for region-specific risk assessment protocols in Saudi dental settings, emphasising medical history screening, medication review, and targeted preventive strategies for medically compromised patients.
